# Comprehensive analysis of cytokine adsorption properties of polymethyl methacrylate (PMMA) membrane material

**DOI:** 10.1007/s10047-022-01323-6

**Published:** 2022-03-17

**Authors:** Tatsuya Kishikawa, Hiroaki Fujieda, Hirokazu Sakaguchi

**Affiliations:** Advanced Materials Research Laboratories, Toray Industries, Inc, 2-1 Sonoyama 3-chome, Otsu, Shiga 520-0842 Japan

**Keywords:** Adsorption, Cytokine, Hemofilter, Polymethyl methacrylate

## Abstract

In acute kidney injury caused by sepsis (septic AKI), excessive production of inflammatory mediators is believed to be involved in deterioration of the disease. Renal replacement therapy using a polymethyl methacrylate (PMMA) membrane hemofilter improves the pathological condition of septic AKI by adsorbing and removing inflammatory cytokines. However, the adsorption properties of the PMMA membrane are unclear. In this study, we comprehensively analyzed the adsorption of 48 different cytokines in human plasma to PMMA and polysulfone (PS) membranes. Seventy-nine percent (38/48) of the cytokines were adsorbed more efficiently to the PMMA membrane than the PS membrane, which indicates that the PMMA membrane has high cytokine adsorption ability. The adsorption rate tended to be higher for the cytokines with lower molecular weight, and there was a significant correlation between the molecular weight of the cytokines and the adsorption rates. Electron microscopy showed that the PMMA hollow fiber membrane had a uniform internal structure from the inner to the outer layers of the membrane and had nano-pores inside the membrane that may have contributed to the adsorption of proteins with a specific molecular weight range. The clinical efficacy of a PMMA membrane hemofilter with cytokine adsorption properties against septic AKI needs further investigation including the evaluation of filtration performance of the hemofilters.

## Introduction

Acute kidney Injury (AKI) is a frequent complication in critically ill patients with high morbidity and mortality. Sepsis is the most common cause of AKI in intensive care units, and it is observed in 40–50% of patients with AKI [1]. In septic AKI, various inflammatory mediators are involved in the deterioration of the disease state, and control of these mediators is a possible therapeutic approach. One approach to regulate mediators in the blood of AKI patients is renal replacement therapy (RRT). The main purpose of RRT is water removal, electrolyte correction, and removal of low molecular weight uremic toxins. Removal of mediators, such as inflammatory cytokines, also contributes to the improvement of the disease state.

To enhance the efficiency of the removal of inflammatory mediators, a variety of RRT modalities have been evaluated, including high-volume hemofiltration (HVHF), very high-volume hemofiltration (VHVHF), and high cut-off (HCO) membranes. However, these attempts have not led to significant improvements in therapeutic efficacy, suggesting that blood purification using the principles of filtration and dialysis was not effective for the enhancement of mediator removal. [2–4]. Removal of mediators by membrane adsorption has recently attracted attention as an alternative [5, 6]. Various polymers, such as polysulfone (PS), cellulose acetate, and polyacrylonitrile, are used for the hollow fibers of RRT hemofilters, and each has different characteristics. Polymethyl methacrylate (PMMA) is a unique membrane material with protein adsorption function that reportedly adsorbs various inflammatory cytokines [7, 8]. However, the degree of adsorption depends on the type of cytokine. Additionally, the relationship between the molecular properties of cytokines and adsorption on PMMA membranes has not been clarified.

The purpose of this study was to comprehensively analyze the adsorption of different cytokines to a PMMA membrane and clarify the characteristics of protein adsorption compared with a PS membrane.

## Materials and methods

### Cytokine adsorption

In this in vitro study, two different membrane hemofilters were used; a PMMA membrane hemofilter (Hemofeel CH-W1.8, Toray Industries, Inc., Tokyo, Japan) and PS membrane hemofilter (Hemofeel SNV-1.0, Toray Industries, Inc.). The outer cylinder case of each filter was cut and the hollow fibers inside were removed and used. A 105-cm-long PMMA hollow fiber and a 75.5-cm-long PS hollow fiber, both with a 0.0228-cm^3^ volume, were further prepared for the adsorption experiment. This volume matches the ratio of plasma volume to hollow fiber volume in a clinical setting. The hollow fibers were shredded into approximately 1-mm lengths [9], washed with normal saline, and used for the adsorption experiments.

Human plasma containing cytokines were prepared as follows. A freeze-dried, standard cytokine mixture containing 48 different cytokines and growth factors (Bio-Plex-pro 48 Plex Kit, Bio-Rad Laboratories, Inc., Hercules, CA, USA) was reconstituted with 0.25 mL citrated human plasma (Tennessee Blood Services, Memphis, TN, USA) and vortexed for 5 s to mix completely. This stock solution was diluted tenfold with the same human plasma and was further used as the cytokine mixture solution for the adsorption experiments. The molecular weight, isoelectric point, and concentrations used for the adsorption experiments of each cytokine are listed in Table 1.

**Table 1:** Characteristics of cytokines

Cytokine	Molecular weight (Da)	pI	Concentration before adsorption (pg/mL)	Cytokine	Molecular weight (Da)	pI	Concentration before adsorption (pg/mL)
SDF-1α	7610	9.7	1800	FGF basic	16,408	9.6	2887
MIP-1 α	7717	4.8	114	IL-1ra	17,126	5.4	10,501
MIP-1β	7819	4.8	440	IL-1β	17,377	5.9	279
RANTES	7851	9.2	1529	IL-7	17,387	8.7	2446
GROa	7865	9.5	6113	IL-1α	18,048	5.3	5417
Eotaxin	8365	9.9	259	IL-18	18,217	5.0	946
IP-10	8646	10.2	3498	SCF	18,529	5.1	2583
MCP-1	8685	9.4	864	G-CSF	19,058	5.4	5183
IL-8	8922	9.2	976	VEGF	19,082	7.9	8825
MCP-3	8956	9.7	425	IFN- α2	19,241	6.0	1906
CTACK	10,150	9.1	1410	LIF	19,716	9.3	5597
MIG	11,725	10.3	2456	IL-6	20,813	6.2	450
MIF	12,345	8.2	3340	PDGF -bb	24,589	9.4	4100
IL-16	12,422	5.1	2252	SCGF-β	24,741	4.7	176,015
IL-15	12,774	4.5	17,556	β-NGF	26,989	9.0	483
IL-5	13,149	7.0	6607	TRAIL	28,401	7.7	1345
IL-13	13,292	8.7	589	IL-2ra	28,447	6.4	1877
IL-9	14,121	9.0	1131	IL-12 p40	34,697	5.4	23,183
GM-CSF	14,478	5.2	593	IL-10	37,295	7.6	1553
IL-4	14,963	9.3	261	TNF-α	52,058	7.0	3436
IL-3	15,091	7.1	164	TNF-β	55,984	8.9	1287
IL-17A	15,124	8.6	3319	M-CSF	56,927	5.1	1041
IL-2	15,418	7.1	2889	IL-12 p70	57,184	5.2	2027
IFN-g	16,177	9.5	1816	HGF	79,609	7.6	13,142

*pI* isoelectric point, *SDF-1α* stroma cell-derived factor-1α, *MIP-1α* Macrophage inflammatory protein-1α, *MIP-1*β Macrophage inflammatory protein-1β, *RANTES* Regulated on activation, normal T cell expressed and secreted, *GROa* growth-regulated oncogene α, *IP-10* Interferon-inducible protein-10, *MCP-1* Monocyte chemoattractant protein-1, *IL-8* Interleukin 8, *MCP-3* Monocyte chemoattractant protein 3, *CTACK* Cutaneous T cell-attracting chemokine, *MIG* Monokine induced by γ interferon, *MIF* Macrophage migration inhibitory factor, *IL-16* Interleukin 16, *IL-15* Interleukin 15, *IL-5* Interleukin 5, *IL-13* Interleukin 13, *IL-9* Interleukin 9, *GM-CSF* Granulocyte macrophage colony-stimulating factor, *IL-4* Interleukin 4, *IL-3* Interleukin 3, *IL-17A* Interleukin 17A, *IL-2* Interleukin 2, *IFN-g* Interferon γ, *FGF basic* Fibroblast growth factor basic, *IL-1ra* Interleukin 1 receptor antagonist, *IL-1*β Interleukin 1β, *IL-7* Interleukin 7, *IL-1*α Interleukin 1α, *IL-18* Interleukin 18, *SCF* stem cell factor, *G-CSF* Granulocyte colony-stimulating factor, *VEGF* Vascular endothelial growth factor, *IFN-*α*2* Interferon α2, *LIF* Leukemia inhibitory factor, *IL-6* Interleukin 6, *PDGF-bb* Platelet-derived growth factor bb, *SCGF-*β Stem cell growth factor β, β*-NGF* β-Nerve growth factor, *TRAIL* TNF-related apoptosis-inducing ligand, *IL-2ra* Interleukin 2 receptor antagonist, *IL-12 p40* Interleukin-12 p40, *TNF*α tumor necrosis factor α, *TNF*β tumor necrosis factor β, M-CSF Macrophage colony-stimulating factor, *IL-12 p70* Interleukin-12 p70, *HGF* hepatocyte growth factor

**Fig. 1:**
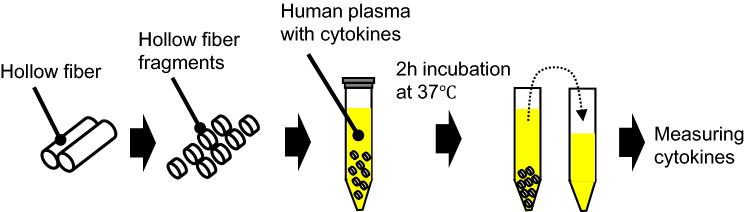
Schematic procedure of in vitro adsorption experiment of cytokines on hollow fiber fragments

A schematic diagram of the experimental procedure is shown in Fig. 1. The hollow fiber fragments described above were suspended in 1080 μL of the cytokine solution and allowed to react for 2 h at 37 °C. During the reaction, the tube was slowly stirred using a rotator to maintain uniform contact between the plasma solution and hollow fiber fragments. After the reaction, the tubes were stored without stirring for 1 min to allow the hollow fibers to settle, and the plasma at the top was collected with a pipette and transferred to another tube to measure the cytokines. The same cytokine solution was incubated without hollow fiber fragments and used as a blank control.

The cytokine measurements were performed using the Bio-Plex-pro 48 Plex Kit according to the manufacturer's protocol. The adsorption rate of each cytokine was calculated by the following formula.

$${\text{Adsorption rate }}\left( \% \right)\, = \,{1}00{-}\left( {{\text{Concentration after incubation with hollow fiber fragments}}/{\text{Concentration after incubation of blank control}}} \right)\, \times \,{1}00$$

### Electron microscopy

The hollow fibers were frozen by immersing them in liquid nitrogen. The samples were then folded to expose the fiber cross section and vacuum dried to produce dried samples. A thin film of platinum was then created on the sample surface by sputtering to observe the sample. The dried samples were observed with a scanning electron microscope (SEM–EDX Type H, Hitachi High-Tech Corp., Tokyo, Japan).

### Statistical analysis

The results of adsorption experiments were expressed as the mean ± standard deviation (SD) of four independent experiments. Student’s *t* test was used to compare data between two groups and *p* < 0.05 was considered statistically significant. The correlation between the adsorption rate and molecular weight or isoelectric point of the cytokines were tested by Pearson's correlation coefficient analysis.

## Results

**Fig. 2:**
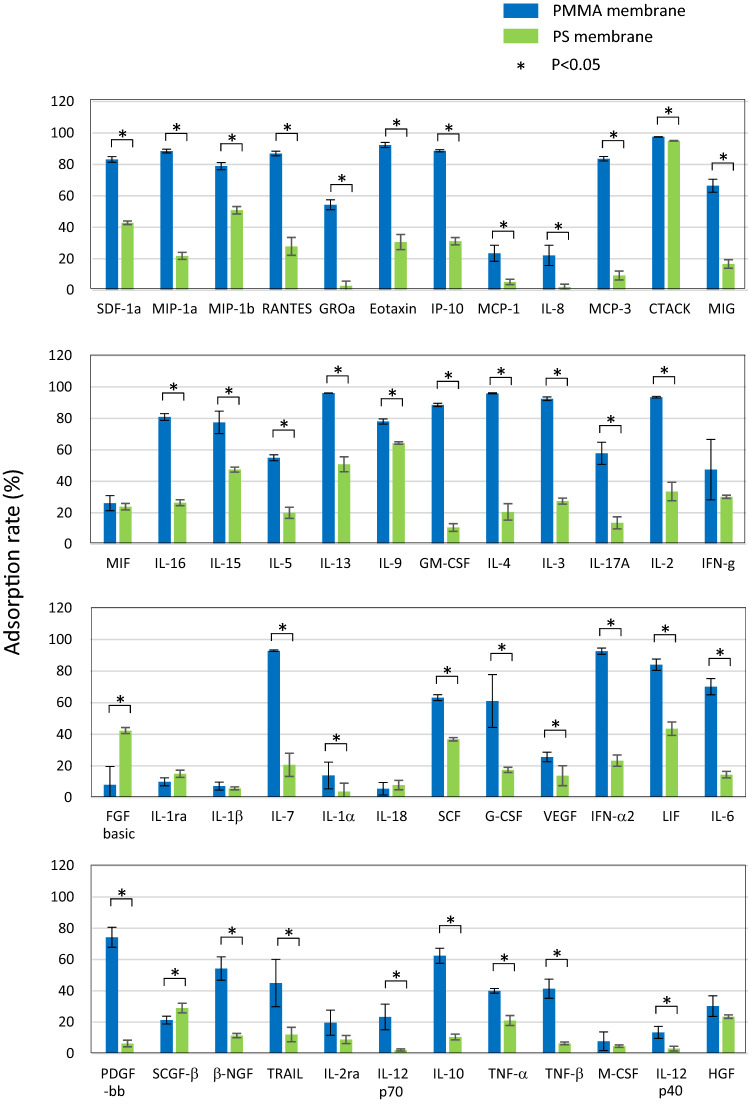
Adsorption rate of each cytokine. Human plasma containing 48 different cytokines was incubated with fragments of PMMA (blue bar) or PS (green bar) hollow fiber fragments for 2 h. The adsorption rate was calculated as described in the Materials and Methods, and depicted as percent adsorption

Figure 2 shows the adsorption rates of cytokines incubated with hollow fiber fragments of the PMMA and PS membranes for 2 h at 37 °C. Adsorption experiments were performed four times for each membrane, and the mean values and standard deviations are shown. The PMMA membrane generally showed a high adsorption rate. However, the adsorption rates varied depending on the type of cytokine, from less than 10% to more than 90%. Alternatively, adsorptions to the PS membrane were generally low. When comparing the adsorption rate of PMMA and PS membranes for each cytokine, PMMA showed higher adsorption properties than PS for 38 of the 48 cytokines, and PS showed higher adsorption for only two (FGF basic, and SCGF-β) of the 48 cytokines. For eight cytokines, there were no significant differences between PMMA and PS.

**Fig. 3:**
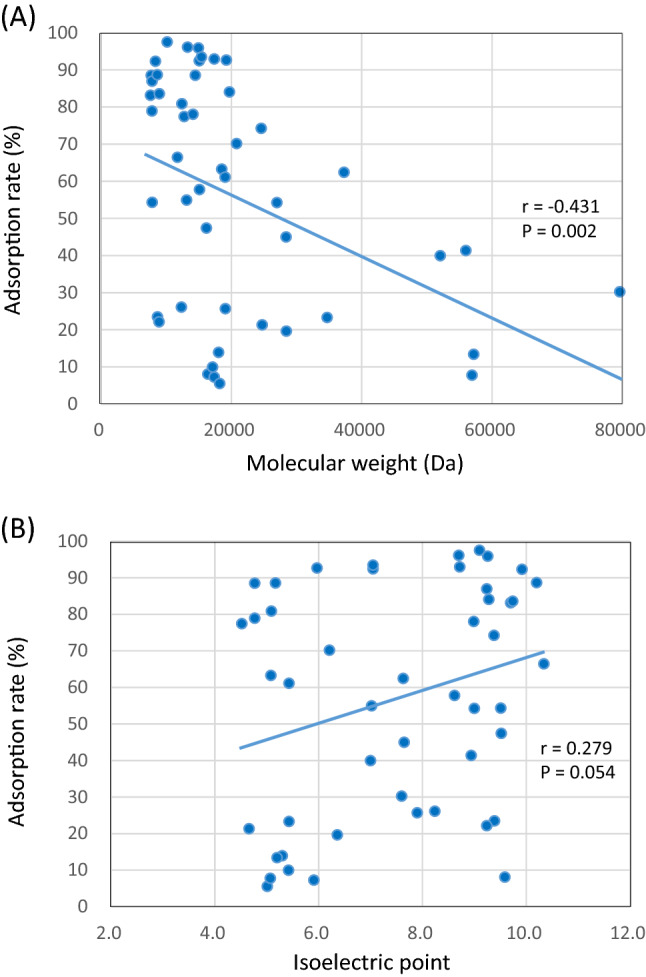
Correlations between the adsorption rate of cytokines to the PMMA membrane and (**A**) molecular weight or (**B**) isoelectric point of the molecules

To examine which characteristics of the cytokines are important for adsorption to the PMMA membrane, the relationships between the molecular weight and adsorption rate, and between the isoelectric point and adsorption rate were examined (Fig. 3). For the molecular weight, a significant correlation was observed with higher adsorption rates for smaller molecular weight cytokines. For the isoelectric point, there was no obvious correlation to the adsorption rate, which suggests that the molecular size is more important than the charge of the molecule for adsorption to the PMMA membrane.

**Fig. 4:**
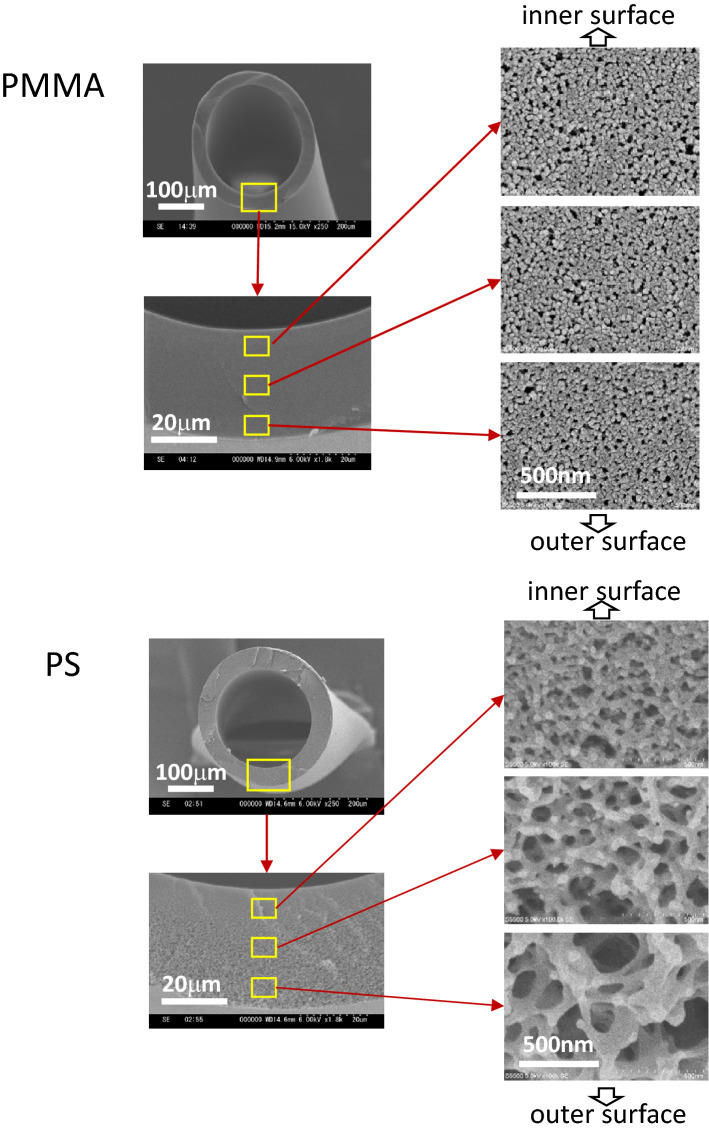
Scanning electron microscope images of the PMMA and PS hemofilter hollow fiber membranes

The internal structure of the hollow fiber membranes was examined by scanning electron microscopy (Fig. 4). The PS membrane had an asymmetric structure with a dense inner surface and a sparse outer surface, while the PMMA hollow fiber had a structure with uniform density from the inner to outer surface with nano-pores, which confirms that the structures of the two membranes were different.

## Discussion

In this in vitro study, the adsorption of 48 different cytokines to a PMMA hollow fiber membrane was evaluated; more than half of the cytokines were adsorbed with high efficiency, with an adsorption rate greater than 50%. Alternatively, adsorption to the PS membrane was lower than to the PMMA membrane. Considering the correlation between molecular weight and the adsorption to the PMMA membrane, a significant correlation was observed with a higher adsorption rates for smaller molecular weight cytokines. Cytokines with a molecular weight range of 10,000–30,000 Da generally adsorbed with high efficiency, while the adsorption rate of cytokines with molecular weight exceeding 30,000 Da tended to be low. There was no clear correlation between the isoelectric point of the cytokine and adsorption rate.

The mechanism of cytokine adsorption to the PMMA membrane is unclear. However, because PMMA is a polymer with hydrophobic properties and does not have a strong electric charge, we speculate that hydrophobic interactions trigger the adsorption. In addition, the PMMA membrane has nano-pores on the surface and inside the membrane, as observed in the electron microscopic image. Cytokines are likely adsorbed by fitting into these nano-pores. The high adsorption rate of cytokines with a molecular weight range of 10,000–30,000 may be attributed to the size of these pores, which fit these molecule sizes.

In addition to PMMA, acrylonitrile/sodium methallyl sulfonate copolymer (AN69) exhibits protein adsorption properties. In the case of AN69, the negative charge inside the membrane contributes to protein adsorption, and cationic substances are adsorbed at higher rates than neutral or anionic substances [7]. This type of adsorption applies to proteins and small molecules. For example, nafamostat mesylate, which is used as an anticoagulant in extracorporeal circulation, has a positive charge and is adsorbed to AN69 membranes [10, 11]. Therefore, an increased dose of the drug should be considered in extracorporeal circulation using AN69 membranes. The membranes of hemofilters may adsorb hazardous substances and various useful components in the blood and drugs including antimicrobials [12, 13]. Therefore, it is necessary to conduct confirmatory studies on the adsorption of various low molecular weight substances for each membrane material.

Cytokine levels are markedly elevated in patients with septic AKI and highly associated with severity of disease. In clinical practice, RRT with a PMMA hemofilter decreases the blood cytokine levels and improves the patient conditions. For example, in a study of 43 patients with septic shock, Nakada et al. reported that RRT using a PMMA hemofilter markedly reduced IL-6 levels in the blood and showed clinical effects, such as a rapid increase in urine output and improvement in hemodynamics [14]. In a recent study using a pig model of septic AKI, treatment with a PMMA hemofilter was more effective in the recovery of kidney function than a PS hemofilter because of inhibition of the complement activation and reduction of renal fibrosis and circulatory inflammatory factors [15]. These results suggest the clinical usefulness of a PMMA hemofilter in septic AKI patients.

This study has several limitations. This study investigated the characteristics of PMMA and PS as membrane materials, and the results may not precisely reflect clinical practice using an actual filter. In addition, the study was conducted using human plasma, which may be different from whole blood. The details of cytokine adsorption characteristics to a PMMA hemofilter in clinical settings and its contribution to the clinical effects on septic AKI need to be validated by large comparative clinical studies including the evaluation of filtration performance of the hemofilters.

## Conclusion

We evaluated the adsorption of 48 different cytokines to hemofilter membranes by in vitro experiments. The PMMA membrane adsorbs more cytokines than a PS membrane, and the adsorption efficiency to the PMMA membrane depends on the molecular weight of the cytokines, which may be reflected by the structure of the membrane. The contribution of cytokine adsorption to a PMMA hemofilter on its clinical efficacy should be investigated in clinical studies.

## References

[1] Peerapornratana S, Manrique-Caballero CL, Gómez H, Kellum JA (2019). Acute kidney injury from sepsis: current concepts, epidemiology, pathophysiology, prevention and treatment. Kidney Int.

[2] Bellomo R, Cass A, Cole L, Finfer S, Gallagher M, Lo S, McArthur C, McGuinness S, Myburgh J, Norton R, Scheinkestel C, Su S, RENAL Replacement Therapy Study Investigators (2009). Intensity of continuous renal-replacement therapy in critically ill patients. N Engl J Med.

[3] Palevsky PM, Zhang JH, O'Connor TZ, Chertow GM, Crowley ST, Choudhury D, Finkel K, Kellum JA, Paganini E, Schein RM, Smith MW, Swanson KM, Thompson BT, Vijayan A, Watnick S, Star RA, Peduzzi P, VA/NIH Acute Renal Failure Trial Network (2008). Intensity of renal support in critically ill patients with acute kidney injury. N Engl J Med.

[4] Lumlertgul N, Hall A, Camporota L, Crichton S, Ostermann M (2021). Clearance of inflammatory cytokines in patients with septic acute kidney injury during renal replacement therapy using the EMiC2 filter (Clic-AKI study). Crit Care.

[5] Moriyama K, Nishida O (2021). Targeting cytokines, pathogen-associated molecular patterns, and damage-associated molecular patterns in sepsis via blood purification. Int J Mol Sci.

[6] Karkar A, Ronco C (2020). Prescription of CRRT: a pathway to optimize therapy. Ann Intensive Care.

[7] Moriyama K, Kato Y, Hasegawa D, Kurimoto Y, Kawaji T, Nakamura T, Kuriyama N, Shimomura Y, Nishida O (2020). Involvement of ionic interactions in cytokine adsorption of polyethyleneimine-coated polyacrylonitrile and polymethyl methacrylate membranes in vitro. J Artif Organs.

[8] Harm S, Schildböck C, Hartmann J (2020). Cytokine removal in extracorporeal blood purification: an in vitro study. Blood Purif.

[9] Karakoç V, Yavuz H, Adil DA (2004). Affinity adsorption of recombinant human interferon-alfa on a porous dye-affinity adsorbent. Colloids Surf A.

[10] Hirayama T, Nosaka N, Okawa Y, Ushio S, Kitamura Y, Sendo T, Ugawa T, Nakao A (2017). AN69ST membranes adsorb nafamostat mesylate and affect the management of anticoagulant therapy: a retrospective study. J Intensive Care.

[11] Nakamura Y, Hara S, Hatomoto H, Yamasaki S, Nakano T, Miyazaki M, Matsumoto N, Irie Y, Ishikura H (2017). Adsorption of nafamostat mesilate on AN69ST membranes: a single-center retrospective and in vitro study. Ther Apher Dial.

[12] Onichimowski D, Ziółkowski H, Nosek K, Jaroszewski J, Rypulak E, Czuczwar M (2020). Comparison of adsorption of selected antibiotics on the filters in continuous renal replacement therapy circuits: in vitro studies. J Artif Organs.

[13] Körtge A, Majcher-Peszynska J, Heskamp B, Wasserkort R, Mitzner S (2021). Antibiotics removal by continuous venovenous hemofiltration with a novel asymmetric triacetate membrane hemofilter: an in vitro study. Blood Purif.

[14] Nakada TA, Oda S, Matsuda K, Sadahiro T, Nakamura M, Abe R, Hirasawa H (2008). Continuous hemodiafiltration with PMMA Hemofilter in the treatment of patients with septic shock. Mol Med.

[15] Stasi A, Franzin R, Divella C, Sallustio F, Curci C, Picerno A, Pontrelli P, Staffieri F, Lacitignola L, Crovace A, Cantaluppi V, Medica D, Ronco C, de Cal M, Lorenzin A, Zanella M, Pertosa GB, Stallone G, Gesualdo L, Castellano G (2021). PMMA-Based Continuous Hemofiltration Modulated Complement Activation and Renal Dysfunction in LPS-Induced Acute Kidney Injury. Front Immunol.

